# Optimization of whole-cell vaccines with CpG/αOX40/cGAMP to strengthen the anti-tumor response of CD4^+^ T cells in melanomas

**DOI:** 10.1007/s00432-022-04117-8

**Published:** 2022-06-24

**Authors:** Xuedan Du, Jinting Wu, Ye Zhao, Bin Wang, Xiaobo Ding, Qiuyan Lin, Yingyu Chen, Jinduo Zhao, Lixiao Liu, Xiaolu Mao, Zhen Fang, Chunhong Zhang, Wenfeng Li

**Affiliations:** 1 Department of Oncology, Lishui Central Hospital, Lishui, Zhejiang People’s Republic of China; 2 Department of Oncology, The First Affiliated Hospital of Wenzhou Medical University, 2 Fuxue Road, Wenzhou, 325000 Zhejiang People’s Republic of China; 3 Department of Obstetrics and Gynecology, The First Affiliated Hospital of Wenzhou Medical University, Wenzhou, Zhejiang People’s Republic of China; 4 Department of Oncology, Ruian City People’s Hospital, Wenzhou, Zhejiang People’s Republic of China; 5 Department of Neurosurgery, The First Affiliated Hospital of Wenzhou Medical University, Wenzhou, Zhejiang People’s Republic of China; 6 Department of Pharmacy, The First Affiliated Hospital of Wenzhou Medical University, Wenzhou, Zhejiang People’s Republic of China

**Keywords:** Whole-cell vaccines, CpG, OX40 agonist, cGAMP, Melanoma

## Abstract

**Methods:**

In this study, we developed a strategy for the prevention and therapy of melanoma using a whole-cell vaccine combined with a CpG/αOX40/cGAMP triple adjuvant. The CpG/αOX40/cGAMP triple adjuvant was used to co-culture melanoma cells in vitro to induce immunogenic death of tumor cells. The mixture of inactivated tumor cells and the triple drug was an optimized tumor whole-cell vaccine, which was injected subcutaneously into mice for tumor prevention and therapy. Furthermore, we analyzed the changes of immune cells in spleen and tumor by flow cytometry and immunohistochemistry, and detected the changes of cytokines after vaccine application by cytometric bead array to explore the specific mechanism of vaccine.

**Results:**

In vaccine prevention and therapy experiments, it was observed that the tumor growth was significantly inhibited in the whole-cell vaccine group, and the survival time of mice was significantly prolonged. Flow cytometry results showed that the proportion of CD4+ T cells and CD8+ T cells in tumor of mice in vaccine group was higher than that in control group, especially the CD4+ T cells.

**Conclusion:**

The optimized vaccine has the unique ability to amplify tumor-specific CD4+ T cells, which improves antitumor sensitivity, and has a significant effect on the prevention and therapy of melanoma mice.

**Supplementary Information:**

The online version contains supplementary material available at 10.1007/s00432-022-04117-8.

## Introduction

Malignant melanoma (MM) is a highly invasive tumor that poses a great threat to human health and life. It is estimated that there are more than 320,000 new cases and more than 50,000 deaths worldwide in 2020 (Sung et al. 2021). For patients with advanced melanomas, traditional therapies, such as surgery, radiotherapy and chemotherapy, are less effective. Immunotherapy is the fourth pillar of cancer treatment (Achkar and Tarhini 2017). In recent years, immunotherapy, especially autologous tumor vaccines, has been considered a promising avenue for cancer treatment because they can actively generate anti-tumor immune responses and produce durable immune memory against recurrence (Cicchelero et al. 2014).

The antigens of autologous tumor vaccines can take a variety of forms, including whole tumor cells (Keenan et al. 2012), tumor cell lysates, and tumor-specific peptides. Using whole tumor cells as an antigen is a very convenient approach to make a vaccine, which is also known as a whole-cell vaccine, without the need for dendritic cells. However, there are indications that live tumor cells are less safe and have poor immunogenicity. Live tumor cells secrete cytokines that suppress the immune response (Chiang et al. 2010). For example, they secrete interleukin 10 (IL-10) (Loercher et al. 1999), which inhibits T cell function, and vascular endothelial growth factor (VEGF), which affects DC cell differentiation and maturation (Dikov et al. 2001). Immunogenic cell death (ICD) induction of tumor cells can stimulate the presentation of tumor antigens to T cells, which plays a crucial role in activating the immune system against tumors (Cicchelero et al. 2014; Kroemer et al. 2013). In addition, tumor vaccines prepared from immunogenically dead tumor cells do not induce spontaneous tumors, which is an ideal antigen for preparation of autologous tumor vaccines. Whole-cell vaccine research has aroused great enthusiasm due to their unique advantages. First, whole-cell vaccines provide all potential antigens, rather than screening specific types of cancer antigens, allowing the immune response to target multiple antigens simultaneously. Thus, they can significantly reduce the chance of tumor immune escape compared to vaccines that use a single epitope (Chiang et al. 2010; Keenan and Jaffee 2012). Second, a whole-cell vaccine is derived from a patient’s own tumor cells, which eliminates the mismatch of human leukocyte antigen (HLA) and avoids rejection; thus, it is relatively safe. Nevertheless, the whole-cell vaccine is a personalized treatment method that needs to be customized for different patients and cannot be widely applied to all patients. In addition, whole-cell vaccine preparation requires surgery or puncture to obtain tumor tissue in advance, and patients who cannot be operated on will not benefit from this therapy method. Overall, whole-cell vaccines are a worthwhile option for many patients with advanced melanomas who meet the criteria.

For tumors with immune escape, vaccination alone is unlikely to be effective, and strategies for improving the therapeutic potential are warranted. Inspired by the anti-tumor treatment model of radiotherapy combined with chemotherapy, researchers have begun to concentrate on a combination of vaccines (Keenan and Jaffee 2012; Tran et al. 2019; Wolfson et al. 2021). The first-generation cancer vaccine-based combination immunotherapy is a vaccine combined with cytokines. With the discovery of immune checkpoints, the second generation of vaccine combination therapy (vaccine combined with immune checkpoints) has begun to gain attention. Studies have shown that both treatment strategies can enhance the efficacy of the original vaccine. As the study progressed, the researchers found that effective treatment strategies require rational multiple combinations of immunotherapy to fully activate the immune system. Therefore, a third generation of combined vaccines has attracted widespread attention, and their treatment strategy is to take the vaccine as the backbone to engage the immune system and combine multiple drugs reasonably to achieve the goal of comprehensively activating immunity (Fabian et al. 2021; Wolfson et al. 2021). In our previous study, we found that the combination therapy of cytosine-phosphorothioate-guanine oligodeoxynucleotides (CpG ODNs), OX40 antibody and cyclic guanosine monophosphate-adenosine monophosphate (cGAMP) showed excellent anti-tumor efficacy in various cell lines, such as TC1, B16 and CT26, and the survival time of mice, after triple adjuvant therapy was significantly prolonged (Cai et al. 2021). Inspired by these findings, we developed a therapeutic strategy combining triple adjuvant therapy with a whole-cell vaccine.

Cytosine-phosphorothioate-guanine oligodeoxynucleotides (CpG ODNs) are toll-like receptor 9 (TLR9) agonists, and CpG ODNs are often used as anti-tumor adjuvants in clinical trials. CpG has shown excellent anti-tumor effects in various animal tumor models, such as melanoma, fibrosarcoma, lymphoma, bladder cancer, colon cancer and lung cancer (Kawarada et al. 2001; Kunikata et al. 2004; Lonsdorf et al. 2003; Sharma et al. 2003). Substantial evidence indicates that CpG is a perfect adjuvant for tumor vaccines. For example, Novakovic et al. found that the combination therapy of CpG with irradiated tumor cells can inhibit tumor growth in B16F1 mice (Novakovic et al. 2007). Experiments by Zhang et al. confirmed that the combined treatment of CpG and HPV peptide-pulsed dendritic cell vaccines can delay the growth of tumors in a TC1 mouse model (J. Li et al. 2016a, b). In addition, CPG as an enhanced adjuvant for HBV vaccines, malaria vaccines, influenza vaccines, and HIV vaccines has also been supported by many experiments (Scheiermann and Klinman 2014).

OX40 (CD134), a member of the tumor necrosis factor receptor (TNFR) family (Watts 2005), is a costimulatory molecule that provides a costimulatory signal similar to CD28. Animal experiments confirmed that local injection of αOX40 around tumors showed outstanding efficacy in mouse models of CT26, MC38, B16 and A20 (Hebb et al. 2018; Makkouk et al. 2015; Sagiv-Barfi et al. 2018). Moreover, αOX40 also plays a significant role in enhancing the efficacy of tumor vaccines. Esteban et al. demonstrated that an OX40 agonist combined with a peptide vaccine inhibits tumor growth by enhancing the immune response of CD4^+^ T cells and prolongs survival in a mouse model of melanoma (Kumai et al. 2017). William et al. found that the combination therapy of an OX40 agonist/CTLA-4 blockade with a HER2 vaccine can eliminate established breast cancer in mice (Linch et al. 2016). Murata et al. showed that the combination of the OX40 antibody and GM-CSF secreting tumor cell vaccine induced a specific CD8^+^ T cell response, thereby inhibiting tumor growth in breast cancer mice (Murata et al. 2006). Helena et al. evaluated the preventive effect of a tumor nano-vaccine combined with an anti-PD-1 antibody and an anti-OX40 costimulatory molecule on melanoma. Nevertheless, this method could not completely prevent the occurrence of tumors but could delay the progression of tumors to some extent (Conniot et al. 2019).

Pathogen recognition receptor (PRR) agonists are currently widely used to optimize the immunogenicity and efficacy of vaccines. As a key link in the cGAS-STING pathway, cyclic guanosine monophosphate-adenosine monophosphate (cGAMP) plays an extremely important role in the innate and adaptive immunity of tumors (Jiang et al. 2020; Ritchie and Li 2020). Somayeh et al. believed that cGAMP and CpG, as adjuvants, can enhance the anti-tumor effect of the HPV vaccine in a TC1 mouse model (Dorostkar et al. 2021). Studies have confirmed that cGAMP as an adjuvant can enhance the immunogenicity of the *Helicobacter pylori* (Hp) vaccine and hepatitis B virus (HBV) vaccine and promote the body’s cellular and humoural immune response (Chen et al. 2020; Ito et al. 2019). Richard et al. found that the combination of cGAMP and saponin could effectively enhance the protective effect of the influenza vaccine in elderly mouse models (Vassilieva et al. 2019).

Herein, we chose the combined treatment strategy of CpG, αOX40 and cGAMP adjuvant combined with a whole-cell vaccine to explore the preventive and therapeutic effects of the vaccine on a mouse model of melanoma. Our objective was to develop a novel therapy for cancer prevention and therapy by providing antigens from the tumor cell itself and triple adjuvants that assist in the comprehensive activation of innate and adaptive immunity. The combined therapy of vaccines and adjuvants has brought new thinking to the immunotherapy of melanoma, which will provide a significant reference for the clinical application of vaccines.

## Materials and methods

### Study design

We believe that whole-cell vaccines combined with triple adjuvant treatment are a promising immunotherapy method. Our general strategy was mainly divided into three parts: evaluation of vaccine preventive efficacy, evaluation of vaccine therapeutic efficacy, and research on the mechanism of vaccine anti-tumor immune response. We established mouse B16F10 models for prevention and therapy to explore the anti-tumor efficacy of the combined vaccine. Furthermore, we investigated the mechanism of the vaccine, including flow cytometry, immuno-histochemical analysis of immune cell changes in the tumors and spleens of mice, and a cytometric bead array (CBA) for the detection of cytokine changes. This study was approved by the Animal Experimental Ethics Committee of Wenzhou Medical University, and the experiment was conducted in strict accordance with the instructions for the care and use of laboratory animals.

### Reagents

CPG-ODN 2395 was provided by Synbio Technologies (Suzhou, China). The agonistic anti-OX40 antibody was provided by BioXcell (West Lebanon, US). 2′3′-cGAMP was purchased from InvivoGen (Shanghai, China). Hyaluronidase type V, deoxyribonuclease type I, and collagenase type IV were purchased from Absin (Shanghai, China), Annexin V-FITC/PI apoptosis detection kit provided by Multi Sciences (Hangzhou, China), Fixation/Permeabilization Kit (Biosciences, USA).

The following monoclonal antibodies (mAbs) were used for flow cytometry: CD45-APC/Cyanine7, CD3-FITC, CD4-APC, CD8a-PE/Cyanine7, NK-1.1-PE, IFN-γ-BV421, 7AAD-PerCP Cy5.5, Annexin V-FITC, and PI-PE. These antibodies and their isotype controls were purchased from Biolegend (California, USA).

### Cell lines and mice

The B16F10 cell line was purchased from the Chinese Academy of Sciences Cell Bank (Shanghai, China). They were cultured in DMEM supplemented with 10% fetal bovine serum (FBS) (Sigma, USA), 100 µg/mL penicillin (Sigma, USA), and 100 µg/mL streptomycin (Sigma, USA) in a humidified incubator at 37 °C with 5% CO_2_.

Six- to eight-week-old female C57BL/6 mice were purchased from Hangzhou Ziyuan Experimental Animal Technology Co., Ltd. One week before the experiment, the mice were placed in an environment at a temperature of 23 ± 2 °C and a relative humidity of 50 ± 10%. The animal room was illuminated for 12 h and was dark for 12 h to simulate the circadian rhythm. In addition, all the mice were fed ultrapure water and clean food.

### Vaccine preparation and animal studies

For vaccine preparation, we sacrificed B16F10 tumor-bearing mice and surgically removed the tumors. Tumor tissue was digested with hyaluronidase type V (Absin, China), deoxyribonuclease type I (Absin, China) and collagenase type IV (Absin, China). After 30 min incubation at 37 °C, single cells were harvested and stored in a refrigerator at – 80 ℃. We utilized an in vitro tumor cell killing assay to prepare the inactivated tumor vaccine. In detail, 3 h before vaccine injection, tumor cell frozen in the – 80 ℃ refrigerator was resuscitated and re-suspended with PBS. To inactivate tumor cells, every 1 × 10^7^ tumor cells were co-cultured with CpG 50 µg, αOX40 30 µg, and cGAMP 10 µg on ice for 3 h. The mixture of tumor cells and triple adjuvant prepared in this way is the inactivated whole-cell tumor vaccine, which can be directly injected subcutaneously or intra-tumoral in mice for prevention and therapeutic purposes.

For the prophylaxis experiment, the mice were divided into three groups. At 14 and 11 days before tumor inoculation, the first group was s.c. injected with 70 µl of PBS into their right abdomens, the second group received a right abdominal s.c. injection of CpG/αOX40/cGAMP triple adjuvant mixture (CpG 50 µg, αOX40 30 µg, cGAMP 10 µg in 70 µl of PBS), and the third group was s.c. injected with a mixture of CpG/αOX40/cGAMP adjuvant and whole-cell vaccine. Two weeks later, 4 × 10^5^ B16F10 cells were injected s.c. into the left abdomens of the mice.

For the therapeutic experiment, 4 × 10^5^ B16F10 cells were s.c. implanted into the left abdomens of the mice. When the tumor volume reached 10–20 mm^3^ (day 7), the mice were randomly divided into three groups as previously described. The injection was given every 2 days three times in total, and the dose of each drug was the same as that in the prophylaxis experiment. We measured the tumor volume of the mice every 2–3 days. Tumor volumes were calculated using the formula Volume = 1/2 (Length × Width^2^). When the volumes of the tumors reached 1500 mm^3^, the mice were sacrificed according to the animal operation guidelines.

### Flow cytometry assay

Annexin V-FITC/PI apoptosis assay kit (Multi Science, China) was used to detect cell death by flow cytometry. Tumor cells were collected after co-incubation with the CpG/αOX40/cGAMP triple adjuvant for 3 h and 24 h. Cells were washed with PBS and re-suspended with a binding buffer in accordance with the manufacturer’s manual, followed by staining with annexin V-fluorescein isothiocyanate and propidium iodide at room temperature in the dark for 15 min. Apoptotic cells were analyzed using a flow cytometer (Beckman Coulter, USA).

For the prophylaxis experiment, peripheral blood and spleen of mice were prepared for flow cytometric analysis. For the therapeutic experiment, the spleens and tumors of the mice were resected 3 days after the third vaccination. The spleen was ground and filtrated into single cell suspension. Tumors were minced prior to enzymatic dissociation using Hyaluronidase type V (Absin, China), Deoxyribonuclease Type I (Absin, China) and Collagenase Type IV (Absin, China). Then the tumor single cell suspension was obtained by filtration. Erythrocytes in peripheral blood, spleen single cell suspension and tumor single cell suspension were lysed with 0.2% NaCl and 1.6% NaCl solutions. According to the supplier’s instructions, cells were stained with CD45-APC/Cyanine7, CD3-FITC, CD4-APC, CD8a-PE/Cyanine7, NK-1.1-PE on ice for 30 min protected from light. Then cells were washed once in low cytometry staining buffer. 7AAD (Biolegend, USA) was added before flow cytometry analysis. The proportion of CD4^+^ cells and CD8^+^ cells among CD45^+^ cells was determined using flow cytometry. For intracellular staining, prior fixation and permeation with a fixation/permeabilization kit are required, depending on the manufacturer's protocol. 

### Cytokines assay

The peripheral blood of the mice was collected and placed in a tube without anticoagulant, placed at room temperature for 2 h, and centrifuged for 20 min (4 °C 3000*G*), and the supernatant was collected. Centrifugation was repeated once until clarified serum was obtained. A cytometric bead array system (BD Accuri C6, Germany) was used to detect the amount of IL-6, IL-10 and IFN-γ in the serum of mice. The experiments followed the manufacturer’s instructions.

### Immunohistochemistry (IHC) assay

Mice were euthanized, and the spleen and tumor tissues of the mice were removed and soaked overnight in 4% formalin. The tissues were fixed with ethanol and xylene at a gradient concentration and then embedded in paraffin wax. A 4–5 mm tissue section was laid flat on adhesive glass slides. Tumor sections were treated with endogenous peroxidase blocking buffer (Beyotime, China) for 10 min to reduce endogenous peroxidase activity. Slides were incubated overnight with rabbit anti-CD3 primary antibody (1:400, Cell Signalling Technology, USA) at 4 °C. Goat anti-rabbit secondary antibody was incubated for 40 min at room temperature. Finally, the sections were visualized with diaminobenzidine (DAB) and haematoxylin staining successively. The state of the section could be observed and imaged under a microscope.

### Statistical analysis

All statistical analyses were performed by GraphPad Prism 7.0. A two-way ANOVA was conducted to compare tumor growth curves among groups. Statistical significance among groups was determined by one-way ANOVA and Tukey’s post hoc test. The Kaplan–Meier method was used for mouse survival analysis, and the log-rank test (Mantel–Cox) was employed to calculate *P* values. *P* values < 0.05 were considered significant. (*p < 0.05; **p < 0.01, and ***p < 0.001)

## Results

### Production and safety confirmation of the whole-cell vaccine

**Fig. 1:**
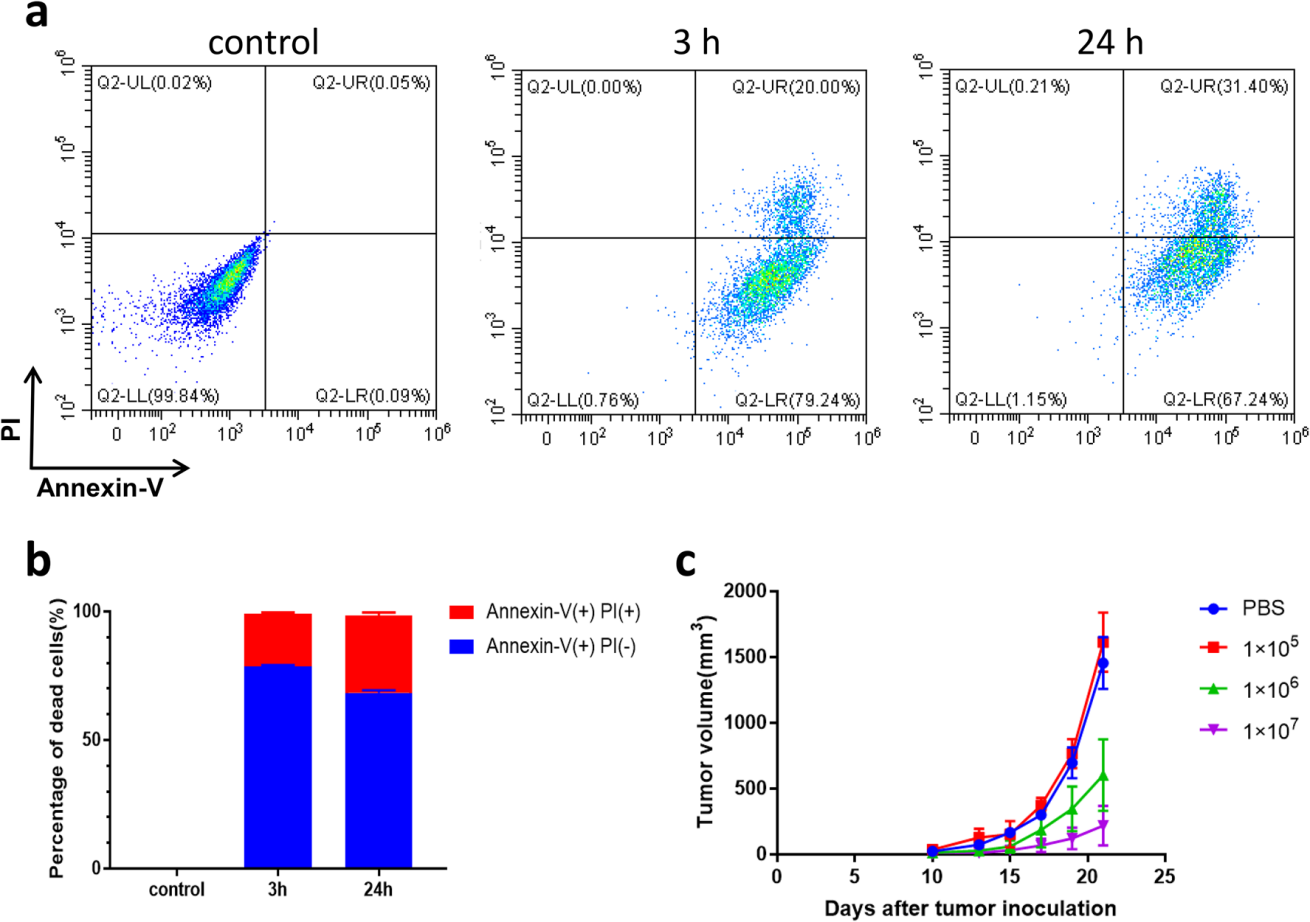
**a** Flow cytometry was performed to detect the survival of tumor cells after 3 h and 24 h co-culture of tumor single cell suspension with triple adjuvants. **b** Flow cytometry quantified the percentage of dead cells. **c** Tumor volume curve. Three different whole-cell tumor vaccines were prepared by co-incubating 1 × 10^5^, 1 × 10^6^, 1 × 10^7^ tumor cells with 50ug CpG, 30ug OX40 and 10ug cGAMP for 3 h in vitro. The whole-cell vaccine was subcutaneously injected into the right abdomen of mice on days 14 and 11 before tumor cell inoculation. Two weeks later, 2 × 10^5^ B16F10 cells were subcutaneously injected into the left abdomen of the mice. Tumor volume changes in mice were monitored every 2–3 days

We extracted tumor tissues from B16F10 tumor-bearing mice under aseptic conditions and prepared them into a single cell suspension. Then CpG/αOX40/cGAMP triple adjuvant was co-incubated with tumor single cell suspension for 3 h and 24 h in vitro, and the survival status of tumor cells after co-incubation was detected. Flow cytometry results showed that tumor cells were completely inactivated after 3 h and 24 h co-incubation with triple adjuvant (Fig. 1a, b), which was consistent with the trypan blue assay (Supplementary Fig. S1). Immunogenically dead tumor cells are not only safe but can also enhance the immune response of the vaccine, which is an ideal vaccine for our experiment. Therefore, we chose co-incubation for 3 h as the standard process for vaccine preparation.

### Assessment of the preventive effect of the whole-cell vaccine

The tumor antigens provided by inactivated tumor cells are the key to the efficacy of whole-cell vaccines. Vaccines with different tumor antigen doses have different anti-tumor effects. To explore the most suitable dose of tumor antigen for whole-cell vaccine preparation, we prepared three whole-cell tumor vaccines containing 1** × **10^5^, 1** × **10^6^ and 1** × **10^7^ tumor cells, respectively. The mice were divided into four groups. Different whole-cell vaccines were subcutaneously injected into the right abdomen of mice in each group. A second shot of the vaccine was given at the same site 2 days later. After 14 days, 2** × **10^5^ tumor cells were subcutaneously injected into the left abdomen of the mice. Tumor size was measured every 2–3 days. And the tumor volume curve showed that the whole-cell vaccine produced from 1** × **10^7^ tumor cells had the best anti-tumor effect (Fig. 1c). Therefore, we selected 1** × **10^7^ tumor cells as the standard antigen dose for vaccine preparation.

**Fig. 2:**
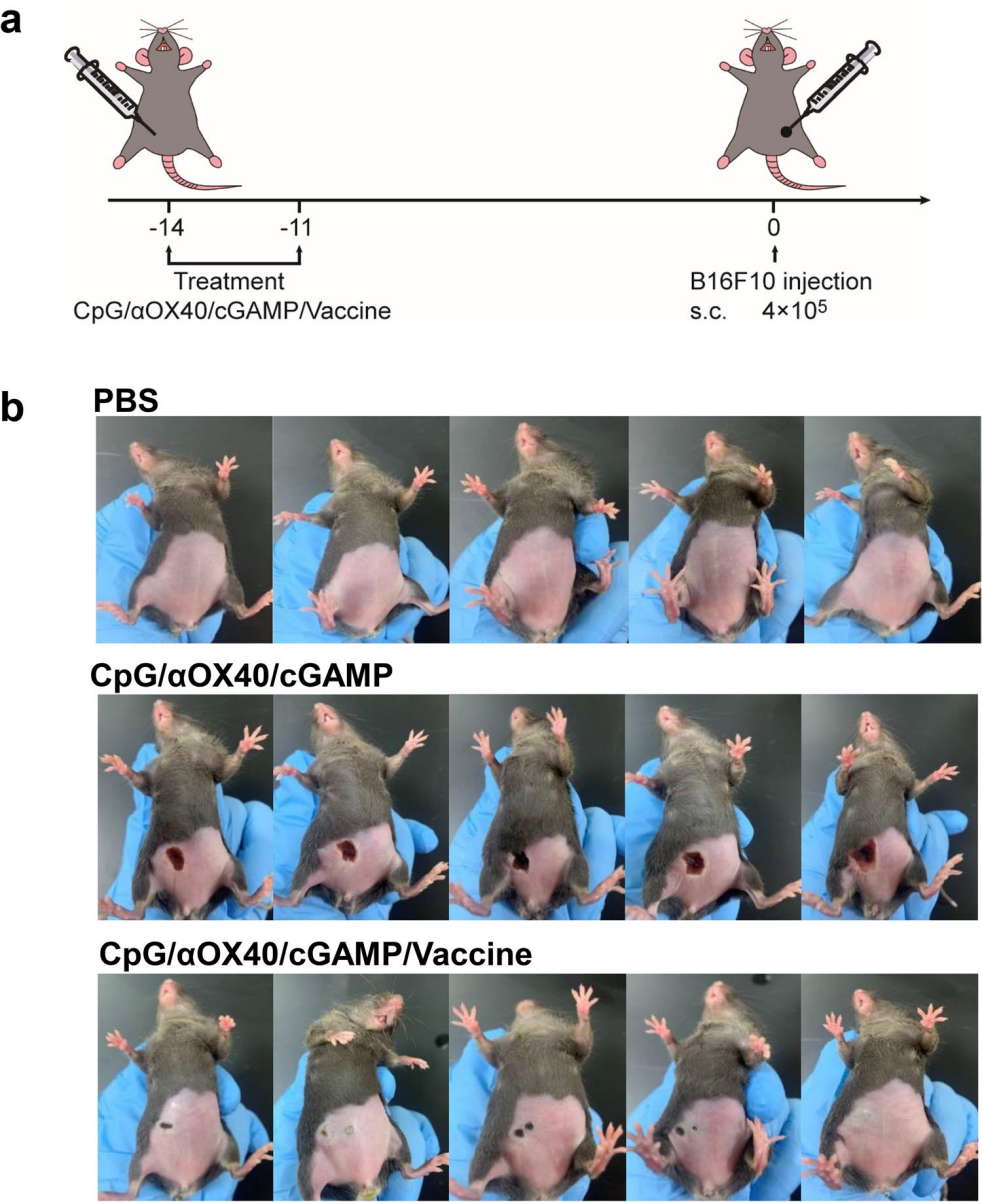
Schematic illustration of the vaccine prophylaxis experiment and photographs of mice after vaccination. **a** Flow chart of prevention experiments. Vaccine was s.c. injected into the right abdomen of mice 14 and 11 days before tumor cell injection (a mixture of CpG 50ug, αOX40 30ug, cGAMP10ug and 1 × 10^7^ whole-cell vaccine co-incubated for 3 h). Two weeks later, 4 × 10^5^ B16F10 cells were s.c. injected into the left abdomen of mice. **b** Pictures of mice 3 days after the second vaccination

The combination dosing schedule for the mice is described in Fig. 2a. The mice were divided into three groups: the control group, CpG/αOX40/cGAMP adjuvant group and combined vaccine group. At 14 and 11 days before tumor cell inoculation, different groups of mice were given the corresponding immunotherapy. Interestingly, we observed severe skin breakage accompanied by brown scabs at the injection site after the second injection in the triple adjuvant group. However, only mild scabs appeared at the injection site of the mice in the combined vaccine group, and some mice even had no skin damage, verifying that our combined vaccine had a high safety and few side effects (Fig. 2b).

**Fig. 3:**
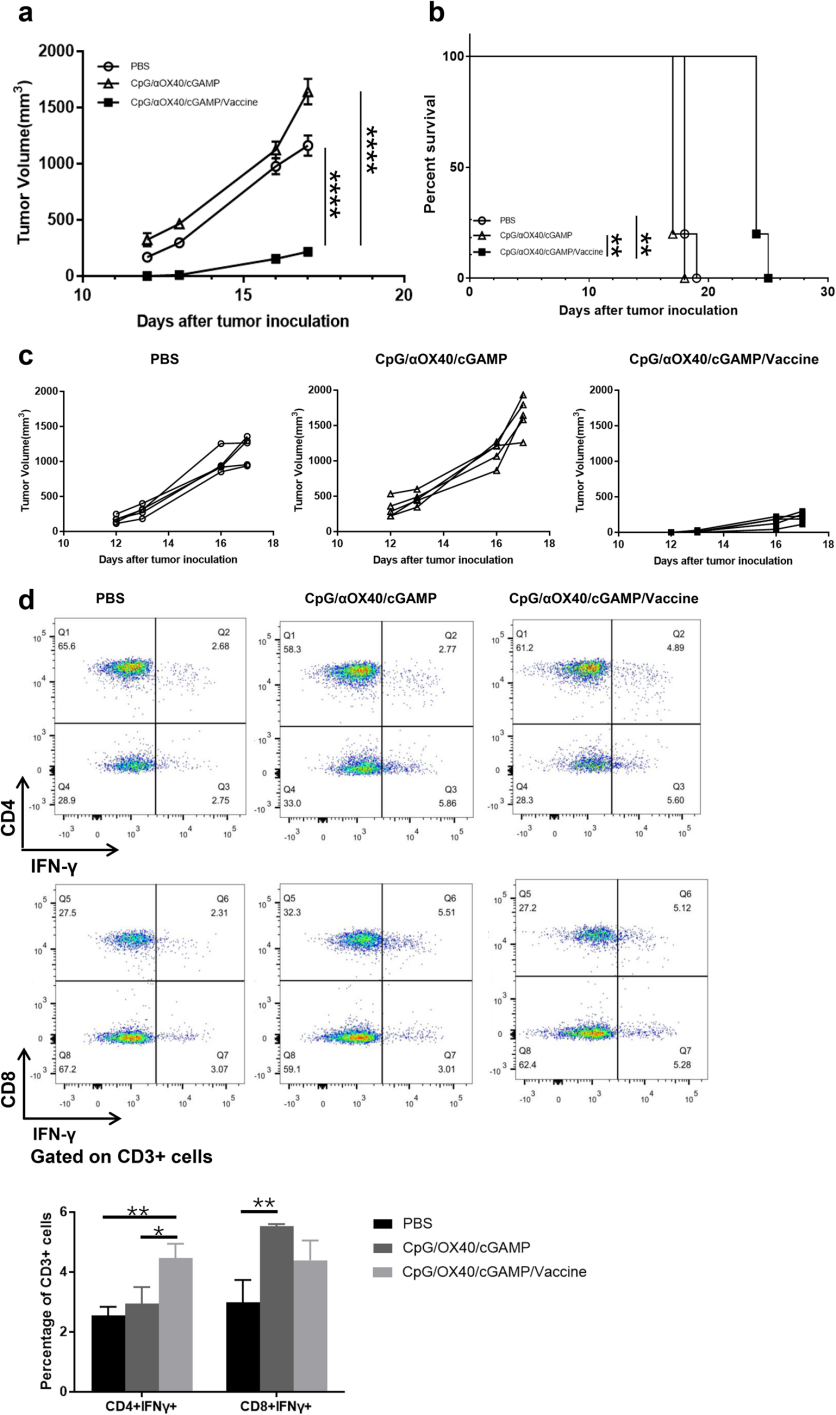
Tumor growth curve and flow cytometry of spleen from vaccine prophylaxis experiments. **a** Tumor growth curves of melanoma model (*n* = 5). **b** The survival of melanoma-bearing mice (mice with tumor volume > 1500mm^3^ were considered dead). **c** Tumor volume curves for each melanoma-bearing mouse. **d** The spleen of mice was collected 10 days after tumor cell injection, and flow cytometry assay was employed to detected the IFN-γ-secreting CD4^+^ /CD8^+^ T cells. For the statistical analysis, there were conducted three independent experiments

Two weeks after vaccination, 4** × **10^5^ B16F10 cells were subcutaneously injected into the left abdomens of the mice. Since tumor volume was not visible to the naked eye for a period of time after the injection of tumor cells, we measured the tumor size of the mice starting from day 12. From the tumor volume diagram of mice, we could determine that the anti-tumor efficacy of the combined vaccine was significantly better than that of the control group and triple adjuvant group, which indicated that the combination of the triple adjuvant and the whole-cell vaccine had a synergistic effect and could simultaneously expand CD4^+^ T cells and CD8^+^ T cells in the body, produce powerful anti-tumor efficacy and prolong the survival time of the mice (Fig. 3a–c). On day 10 after tumor cell injection, the spleens of mice were collected for flow cytometry analysis. As a result, we found that the optimized whole-cell vaccine could effectively promote the proliferation of IFN-γ secreting CD4^+^ T cells (Fig. 3d). This result gives us confidence in the combination strategy and led us to design a therapeutic intervention strategy for the B16F10 tumor-bearing mouse model.

### Assessment of the therapeutic effect of the whole-cell vaccine

**Fig. 4:**
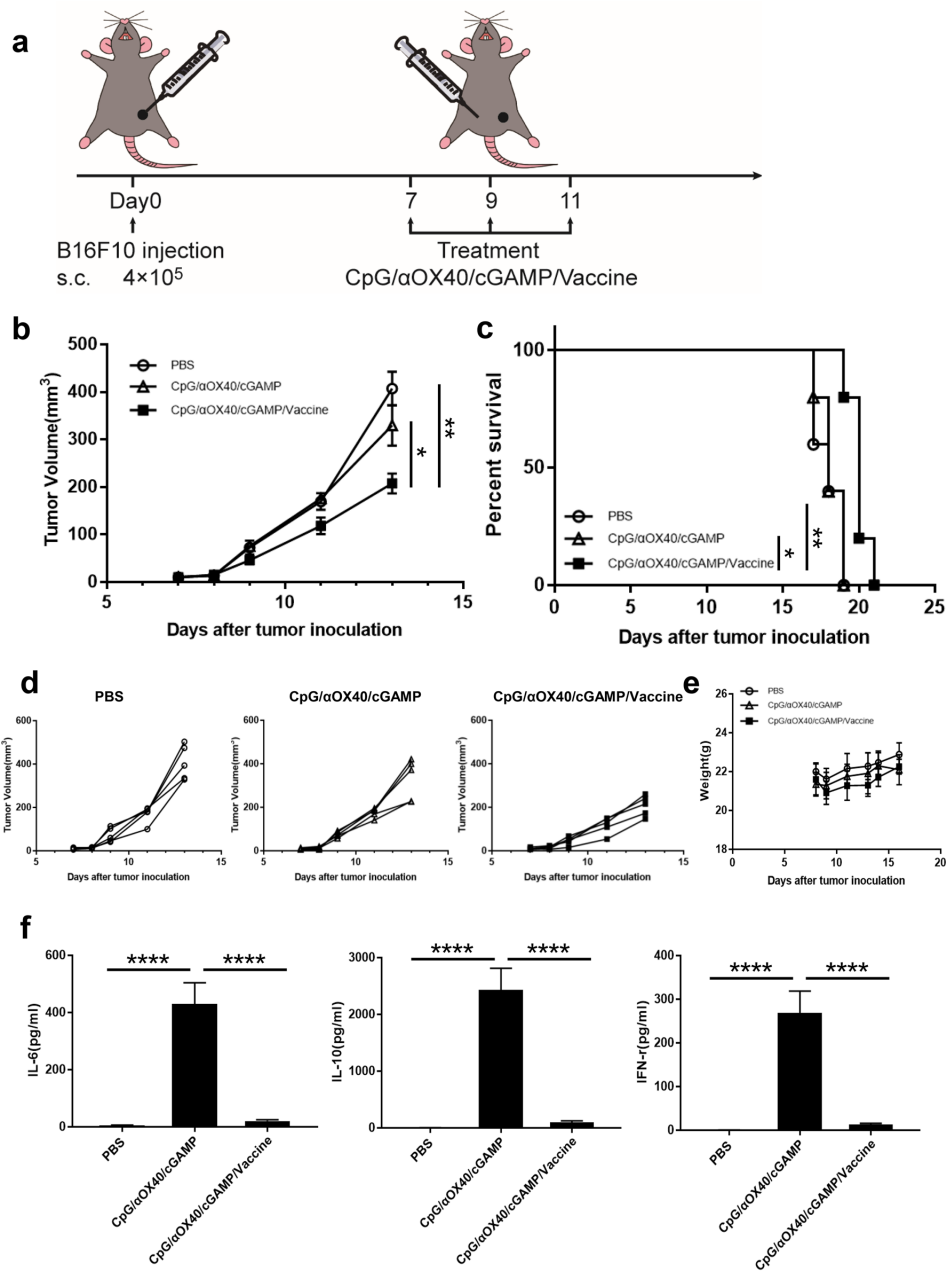
Therapeutic effect of whole-cell vaccine optimized with CpG/αOX40/cGAMP triple adjuvant. **a** Treatment schedule of melanoma-bearing mice. 4 × 10^5^ B16F10 cells were s.c. injected into the left abdomen of six to eight-week-old mice. After the tumor volume reached 10–20 mm^3^ (Day 7), the mice were s.c. injected into the right abdomen with the optimized triple whole-cell vaccine (the preparation method of combined vaccine was the same as the preventive experiment). The vaccination was given once every other day for three times in total. Tumor volume (**b**) and survival (**c**) of melanoma-bearing mice (*n* = 5 mice per group). **d** A tumor volume was measured in each group (The curve shows tumor volume changes within 13 days after tumor cell injection). **e** Changes in body weight of mice during the experiment. **f** Mice were sacrificed on day 14 and their peripheral blood was collected to analyze the secretion of IL-6, IL-10 and IFN-γ in serum

We established a melanoma-bearing mouse model and treated it according to the schedule in Fig. 4a. On the 7th day after tumor cell injection, mice with a tumor volume of 10–20 mm^3^ were screened and randomly divided into three groups for prophylaxis. To investigate the therapeutic effect of the combined vaccine, subcutaneous injection was made to the distal site of the tumor in the mice (right abdomen). The treatment was repeated three times every other day. Tumor size was measured every 2–3 days in each group. Compared with the blank control group and the three adjuvant groups, the combined vaccine group could significantly inhibit tumor growth and prolong the survival time of mice (Fig. 4b–d and Supplementary Fig. S2). In addition, we observed the appearance and behavior characteristics of the mice, and recorded the changes of body weight of mice during the experiment (Fig. 4e). It was found that none of the vaccinated mice exhibited side effects on gross features, such as weight loss, abnormal behavior, accidental death, etc.

**Fig. 5:**
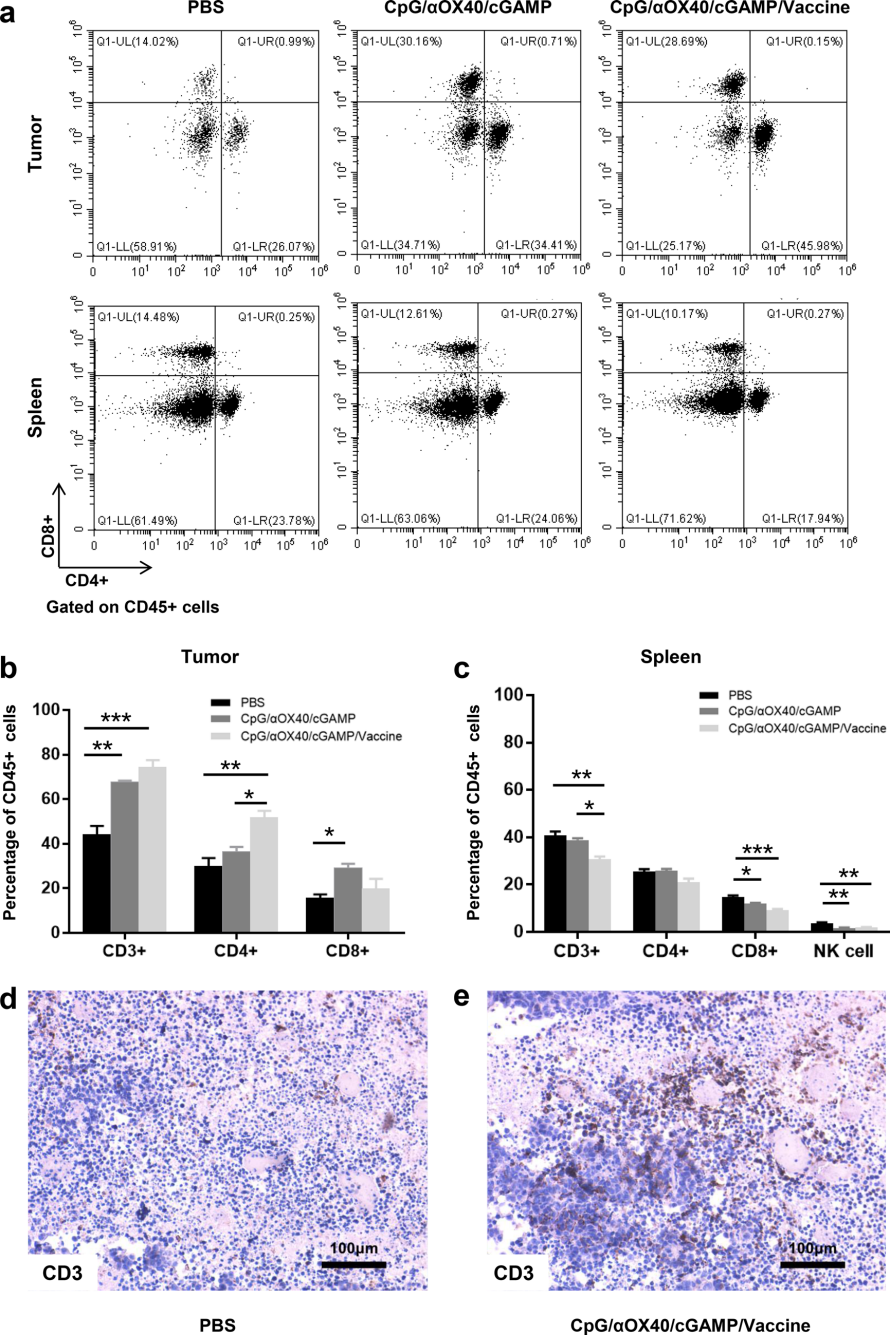
Percentage of relevant T cells in tumor and spleen. **a–c** Mice were sacrificed on day 14, the tumor and spleen of mice was collected for the analyzing of flow cytometry, the results are visualized with a bar graph (The bar graphs represent the quantitative analysis of three independent experiments). **d**, **e** Immunohistochemistry for CD3 shows diffuse infiltration by CD3^+^ T cells in tumor tissue, × 100

It is well known that the difficulty of anti-tumor immunotherapy lies in changes to the immune microenvironment of the tumor. To explore the anti-tumor mechanism of the combined vaccine, the mice were euthanized 3 days after the third vaccination, and the peripheral blood was used for cytokine analysis. The spleen and the tumor were extracted for flow cytometry and IHC analysis. We detected changes in IL-6, IL-10 and IFN-γ in the peripheral blood of the mice. To our surprise, IL-6, IL-10, and IFN-γ were significantly increased in the triple adjuvant group, followed by the triple adjuvant combined with whole-cell vaccine group (Fig. 4f). Besides, we observed severe local skin ulceration at the injection site in mice in the triple adjuvant group, while only minor skin damage was observed in mice in the triple adjuvant combined vaccine group. Based on this, we believe that a cytokine storm occurred in the triple adjuvant group, leading to local skin damage at the injection site. A cytokine storm, also known as cytokine release syndrome (CRS), is an adverse reaction to immunotherapy (Fajgenbaum and June 2020; Tisoncik et al. 2012). CRS was coined to describe a similar syndrome after infusion of muromonab-CD3 (OKT3) (Chatenoud et al. 1991). With the rapid development of tumor immunotherapy, it has been found that CRS can be induced by a variety of immunotherapy methods, such as CAR T cell therapy (Freyer and Porter 2020), systemic IL-2 administration (Panelli et al. 2004), PD-1 inhibitors (Del Bello et al. 2021) and radiation therapy (Barker et al. 2018). CRS is characterized by over-activation of immune effector cells and massive release of various cytokines including IL-6, IL-10 and IFN-γ, which is consistent with our experimental results (Fajgenbaum and June 2020). In addition, the local symptoms of CRS are typical acute inflammation, that is, local redness, fever, pain and loss of function. When inflammation spreads throughout the whole body, severe adverse reactions, such as fever, dyspnea, hypoxemia and even shock, can occur. When it is confined to the skin, it can appear as a local lesion of the skin. On the 3 days after vaccination, the spleens and tumors of the mice were analyzed by flow cytometry. Theoretically, higher T lymphocyte infiltration is associated with stronger tumor suppressor function. As expected, the number of infiltrating lymphocytes in the tumors of mice treated with the triple adjuvant and combined vaccine increased significantly compared to the control group, especially in the combined vaccine group (Fig. 5a, b), which supports the synergistic effect previously observed in the prophylaxis trial and was also confirmed by the immuno-histochemical assay (Fig. 5d, e). Prominent infiltration of CD4^+^ T cells was observed in the tumors of the combined vaccine group mice, while CD8^+^ T cell expansion was more pronounced in the triple adjuvant group (Fig. 5b). This is consistent with what has previously been observed in prophylaxis experiments. Both prophylaxis experiments and therapeutic experiments showed that CpG/αOX40/cGAMP combined with a whole-cell vaccine produced a strong immune response through the activation of CD4^+^ T cells. CD4^+^ T cells can achieve anti-tumor efficacy through a variety of mechanisms, including enhancing antigen presentation and promoting T cell homing and T cell activation (Melssen and Slingluff 2017). In response to the stimulation of the combined vaccine, the immune system of the mice was activated, and T cells proliferated and migrated to the tumor immune microenvironment under the action of chemokines, which displayed a reduced infiltration of T cells in the spleen of the vaccine-treated mice (Fig. 5a, c).

## Discussion

In this study, we optimized a whole-cell vaccine combination with a CpG/αOX40/cGAMP triple adjuvant. This multi-pathway synergistic immunotherapy has shown strong anti-tumor potential in both preventive and therapeutic experiments against melanomas. This work provides an up-to-date overview of the practicality of vaccines with multiple therapeutic combinations and provides a new direction for vaccine preparation.

The recognition of tumor antigens by immune cells is a prerequisite for activation of the immune system. However, the development of tumors is often accompanied by immune escape. Tumor cells can evade immune surveillance by inducing and recruiting inhibitory immune cells, reshaping epigenetics and other immune mechanisms, which makes the immune system of the body unable to recognize tumor cells normally (Liu and Cao 2016; Tang et al. 2020). An effective method to prevent immune escape of tumor cells is to induce immunogenic death of tumor cells. ICD is accompanied by the exposure and release of large damage-associated molecular patterns (DAMPs), which support the recruitment and activation of antigen-presenting cells and promote dying cancer cells with a powerful adjuvant effect (L. Galluzzi et al. 2017; Lorenzo Galluzzi et al. 2020; Schock et al. 2017). Activation of cGAS-STING signals has recently been confirmed to have direct cytotoxic effects on some tumor cells, inducing immunogenic death of tumor cells (Ahn et al. 2018; Wang-Bishop et al. 2020). In normal eukaryotic cells, DNA and cytoplasm are separated. However, in tumor cells, due to the massive replication of cancer cell chromosomes, DNA leaks into the cytoplasm. When cyclic-GMP-AMP synthase (cGAS) senses dsDNA that should not exist in the cytoplasm, cGAMP is generated and then the cGAS-STING pathway is activated. In addition, Pourianazar et al. found that CpG can also exert direct cytotoxic effects by regulating the expression of apoptoses-related genes and the release of cytokines in breast cancer cells (Taghavi Pourianazar and Gunduz 2016). At present, there is no evidence that OX40 antibodies have a direct killing effect on tumor cells in vitro. However, CpG/αOX40 has been proven to have excellent anti-tumor effects, which can promote the release of dsDNA and induce the generation of endogenous cGAMP**,** thereby indirectly activate the cGAS-STING pathway.

A successful anti-tumor vaccine requires complex immune interactions. Whole-cell vaccine therapy alone is unlikely to be effective and rational multi-combination immunotherapy strategies are necessary. In this study, we used CpG/αOX40/cGAMP as an adjuvant to enhance the efficacy of the whole-cell vaccine. The activation of cGAS-cGAMP-STING pathway not only plays a direct killing role in tumor cells, but also plays a crucial role in the activation of innate immunity (Ishikawa and Barber 2008; T. Li et al. 2016a, b; Pepin and Gantier 2017; Woo et al. 2014). Through STING-TBK1-IRF3 signaling axis, cGAMP can not only induce the increased expression of type I interferon (IFN-I) and other interferon-stimulated genes (ISGs), but also promote the release of cytokines, leading to the overall activation of innate immunity. Moreover, anti-tumor immunity is a complex network of biological effects that requires the synergistic activation of innate immunity and adaptive immunity. To maximize the immune effect, we added CpG/αOX40 adjuvant, which has been proved to fully mobilize the innate and adaptive immunity of the body, induce the proliferation and activation of tumor-specific lymphocytes and effectively inhibit the growth of lymphoma, melanoma, cervical cancer and other tumors. As a potential adjuvant, CpG is often used in combination with vaccines, other adjuvants, or immune checkpoints for anti-tumor immunity. CpG binds primarily to TLR9 expressed on B cells and plasmacytoid dendritic cells (pDCs) to initiate an innate immune response and support subsequent activation of adaptive immunity (Iho et al. 2015). The anti-tumor effect of CpG is mainly reflected in its ability to promote the activation and expansion of CD8^+^ T cells while reducing the number of regulatory T cells (Tregs) (Mangsbo et al. 2010; Standley et al. 2007; Wang et al. 2016). Levy et al. found that CpG promoted the expression of OX40 on CD4^+^ T cells (Sagiv-Barfi et al. 2018). Therefore, exogenously added OX40 agonists can activate OX40 on the surface of CD4^+^ T cells and promote the proliferation and differentiation of CD4^+^ T cells. Although each of these ingredients does not work very well as a monotherapy, their combination leads to powerful and widely complementary results. These results provide a strong basis for the design of combination therapies for solid tumors.

The whole-cell vaccine optimized by CpG/αOX40/cGAMP can fully mobilize the immune system and increase the infiltration of lymphocytes (especially CD4^+^ T cells) within the tumor, thus significantly preventing the growth of melanoma and prolonging the survival time of melanoma mice. Moreover, our combined vaccine has high safety, except for slight skin damage at the injection site, and no serious adverse reactions, such as weight loss and fever, occurred in mice. Of course, there are some limitations in our research. The preparation of whole-cell vaccines requires patients to have a resectable tumor sample, and many patients are excluded because they do not meet this requirement. Although the safety and efficacy of the optimized whole-cell vaccine have been demonstrated in mice, the clinical efficacy is not yet clear. In addition, the mechanism by which CpG/αOX40/cGAMP induces tumor cell immunogenic death remains to be further explored.

## Conclusion

In summary, as an immune-activating preventive and therapeutic strategy, CpG/αOX40/cGAMP combined with a whole-cell vaccine has the unique ability to amplify tumor-specific CD4^+^ T cells, which improves anti-tumor sensitivity and long-term tumor recognition. Although currently the combined vaccine as a preventive and therapeutic strategy cannot completely eliminate tumors, there is no doubt that we see a promising future with it. Our preclinical study provides a new therapeutic principle and direction for melanomas that deserves the attention of researchers who focus on melanoma prevention and therapy.

## Supplementary Information

Below is the link to the electronic supplementary material.

Supplementary Figure S1. With single cell suspension as control, the survival status of tumor cells after 3 h and 24 h co-culture of whole-cell vaccine and triple adjuvant was detected by trypan blue assay. (The small bright spots indicate cell survival, and the dim spots indicate cell death) (PDF 277 KB)

Supplementary Figure S2. In the vaccine therapeutic experiment, B16F10 tumor-bearing mice with tumor volume of 10–20 mm^3^ were divided into PBS group, triple drug group and vaccine group for treatment, and the tumor volume of each mouse in these three groups was monitored. The curve shows the change in tumor volume before the death of the mice. (Mice with tumor volume over 1500 mm^3^ were considered dead) (PDF 99 KB)

## Data Availability

The authors declare that the data supporting the findings of this study are available within the paper and its supplementary information files or available from the corresponding author upon reasonable request. Source data are provided with this paper.
