# Supplementary figures and images for: Optimization of whole-cell vaccines with CpG/αOX40/cGAMP to strengthen the anti-tumor response of CD4+ T cells in melanomas
Source: J Cancer Res Clin Oncol. 2022 Jun 24;148(12):3337–50. doi: 10.1007/s00432-022-04117-8 (PMC9587117; doi:10.1007/s00432-022-04117-8)

## Supplementary Figure S1

control

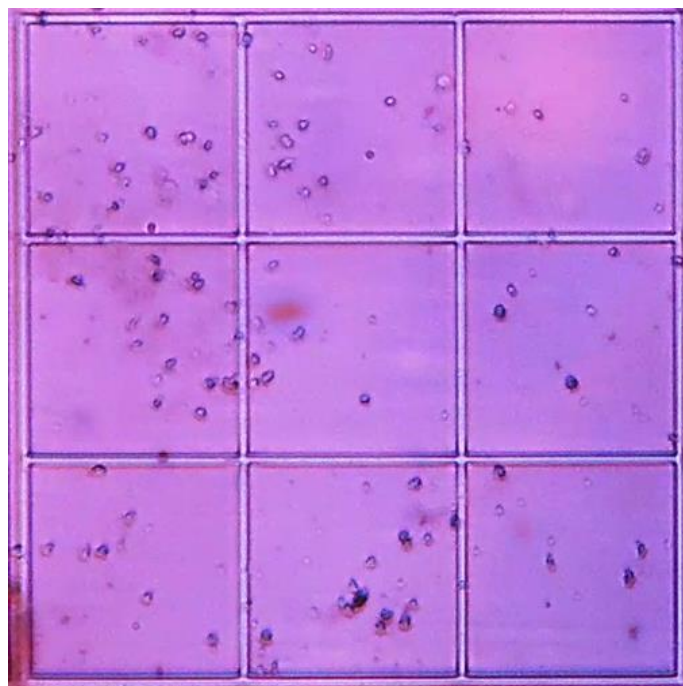

3h

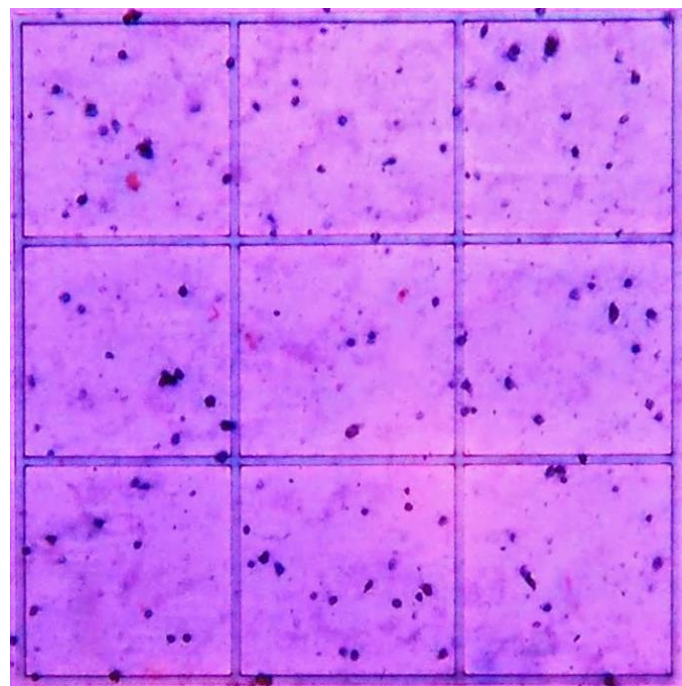

24h

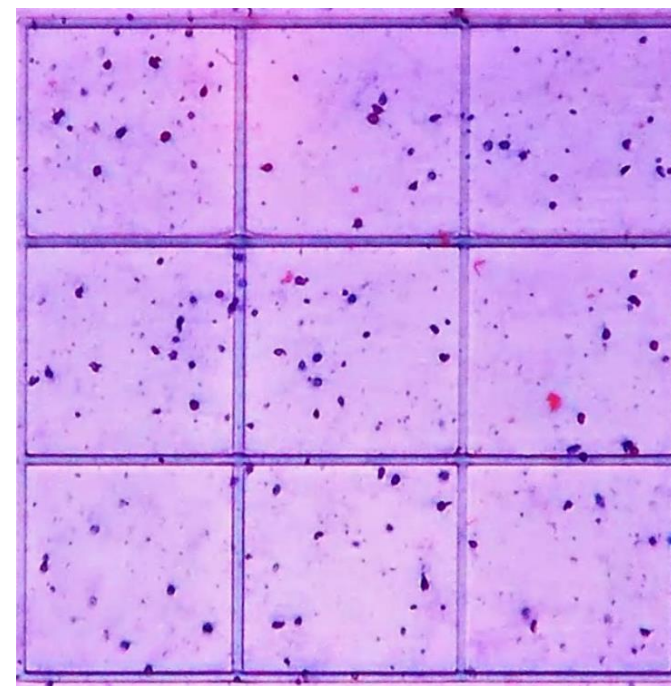

Supplement: Supplementary file 1 — Supplementary Figure S1. With single cell suspension as control, the survival status of tumor cells after 3 h and 24 h co-culture of whole-cell vaccine and triple adjuvant was detected by trypan blue assay. (The small bright spots indicate cell survival, and the dim spots indicate cell death) (PDF 277 KB) [file 432_2022_4117_MOESM1_ESM.pdf]

## Supplementary Figure S2

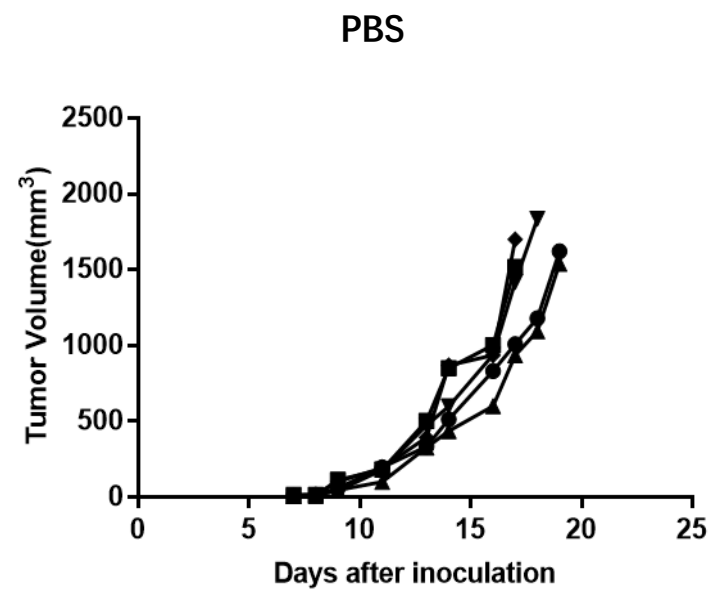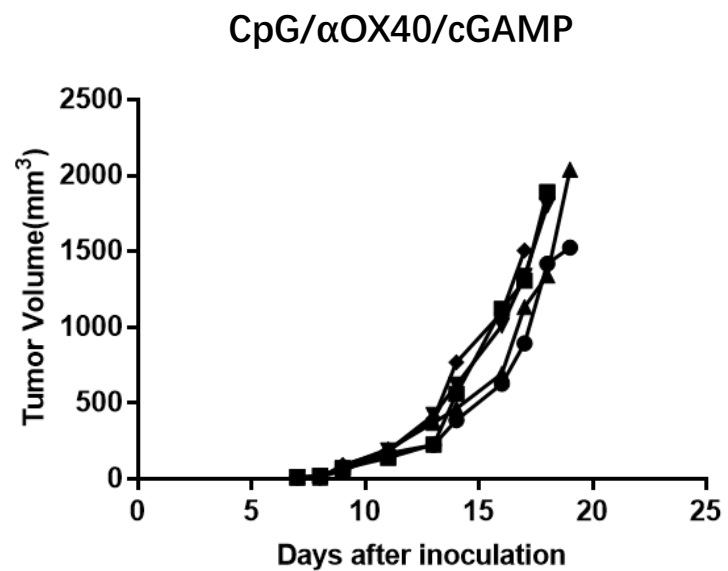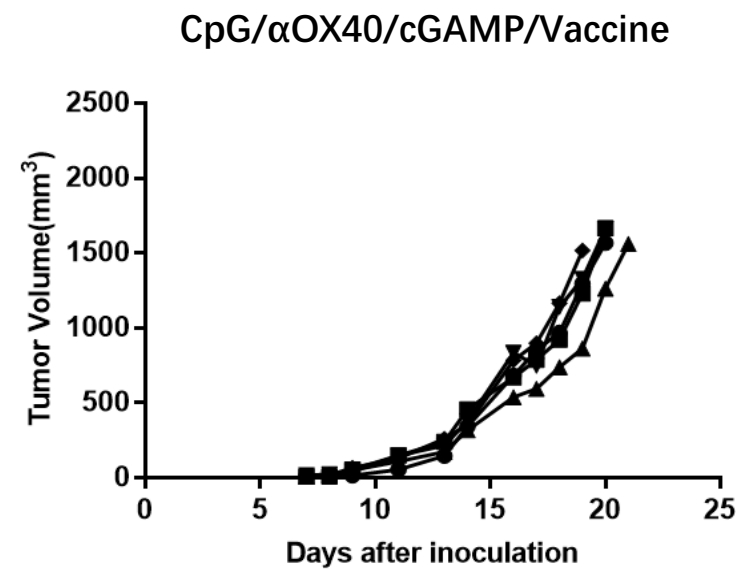

Supplement: Supplementary file 2 — Supplementary Figure S2. In the vaccine therapeutic experiment, B16F10 tumor-bearing mice with tumor volume of 10–20 mm3 were divided into PBS group, triple drug group and vaccine group for treatment, and the tumor volume of each mouse in these three groups was monitored. The curve shows the change in tumor volume before the death of the mice. (Mice with tumor volume over 1500 mm3 were considered dead) (PDF 99 KB) [file 432_2022_4117_MOESM2_ESM.pdf]
